# Ethanol Ablation of Metastatic Lymph Nodes in Patients With Papillary Thyroid Carcinoma—Predictors of Clinical Outcome

**DOI:** 10.1210/clinem/dgaf298

**Published:** 2025-05-23

**Authors:** Pål Stefan Frich, Eva Sigstad, Audun Elnæs Berstad, Else Marie Opsahl, Kristin Holgersen Fagerlid, Krystyna Kotanska Grøholt, Trine Bjøro, Knut Håkon Hole, Liv Ingrid Flinder

**Affiliations:** Division of Radiology and Nuclear Medicine, Department of Radiology, The Norwegian Radium Hospital, Oslo University Hospital, 0424 Oslo, Norway; Institute of Clinical Medicine, Faculty of Medicine, University of Oslo, 0318 Oslo, Norway; Division of Laboratory Medicine, Department of Pathology, Oslo University Hospital, 0424 Oslo, Norway; Division of Radiology and Nuclear Medicine, Department of Radiology, The Norwegian Radium Hospital, Oslo University Hospital, 0424 Oslo, Norway; Division of Cancer Medicine, Department of Surgical Oncology, Section of Breast- and Endocrine Surgery, Oslo University Hospital, 0424 Oslo, Norway; Division of Radiology and Nuclear Medicine, Department of Radiology, The Norwegian Radium Hospital, Oslo University Hospital, 0424 Oslo, Norway; Division of Laboratory Medicine, Department of Pathology, Oslo University Hospital, 0424 Oslo, Norway; Institute of Clinical Medicine, Faculty of Medicine, University of Oslo, 0318 Oslo, Norway; Division of Radiology and Nuclear Medicine, Department of Radiology, The Norwegian Radium Hospital, Oslo University Hospital, 0424 Oslo, Norway; Institute of Clinical Medicine, Faculty of Medicine, University of Oslo, 0318 Oslo, Norway; Division of Radiology and Nuclear Medicine, Department of Radiology, The Norwegian Radium Hospital, Oslo University Hospital, 0424 Oslo, Norway

**Keywords:** *BRAF* ^V600E^, ethanol ablation, lymph node metastasis, papillary thyroid carcinoma, ultrasound-guided therapy, treatment response prediction

## Abstract

**Context:**

Ethanol ablation (EA) is a treatment option in recurrent or persistent metastatic lymph nodes (MLNs) from papillary thyroid carcinoma.

**Objective:**

This work aimed to assess whether ultrasonographic characteristics of the MLN, history of lymph node surgery, aggressive histological subtype, or *BRAF*^V600E^ mutation in the primary tumor predict long-term response from EA.

**Methods:**

Seventy-five patients who received EA at a tertiary referral center were included. We evaluated treatment response from the most recent clinically indicated examination, or a study-specific examination. BRAF^V600E^ analysis and review of histological subtypes in the primary tumor were conducted.

**Results:**

Median interval from initial surgery to follow-up was 119 months (range, 39-471 months). Pure cystic MLN had a better outcome than the solid and partially cystic MLN (13/13, 100% vs 90/121, 74%; *P* = .039). Small MLNs (≤0.5 mL) had a higher response rate compared to larger lesions (71/92, 77% vs 10/19, 53%; *P* = .045). We observed no difference in EA response between patients with or without the *BRAF*^V600E^ mutation (80/99, 81% vs 17/25, 68%; *P* = .181) or an aggressive subtype (22/24, 92% vs 75/100, 75%; *P* = .099) in their primary tumors. EA achieved similar rates of locoregional disease control in neck regions with or without previous lymph node surgery (66% vs 63%, *P* = .825).

**Conclusion:**

EA was highly effective in pure cystic MLNs. Partially cystic or noncystic MLNs over 0.5 mL were less responsive, though many of these MLNs still showed a lasting response. *BRAF*^V600E^ mutation, aggressive histological subtype, or absence of prior lymph node surgery did not negatively affect EA response.

Differentiated thyroid carcinoma is the most common malignancy in the thyroid gland, representing more than 90% of cases; papillary thyroid carcinoma (PTC) constitutes the majority.

Despite the indolent behavior of PTC having excellent prognosis and survival rates, metastatic lymph nodes (MLNs) in the neck are very common (1). MLN are present in 40% to 90% of patients treated with prophylactic central neck dissection (2-4). Metastasis to the lateral neck are rare without evident central MLNs, but present in more than half of patients with confirmed central MLNs (5). At follow-up, a considerable number of patients with PTC experience persistent or recurrent MLNs, despite primary treatment carried out with curative intent (6-8).

Ethanol ablation (EA) was introduced in the early 1990s as a minimally invasive treatment alternative to surgery in persistent or recurrent MLNs from PTC after primary treatment. EA is beneficial in cases for which surgery is not feasible or is associated with higher risk, such as redo surgeries, high comorbidity, or patient preference (9, 10). Several reports have shown good long-term results with durable response in 60% to 95% of treated MLNs (11-18). EA is easily repeatable without technical difficulties, and previous studies have not revealed any serious or persistent side effects or complications (17, 19-21). However, recurrences in the area of EA are of some concern. Although the first reports on EA in this setting showed low recurrence rates, subsequent series with longer follow-up times have reported recurrence rates at the ablation area in 19% to 28% of cases (12, 16, 17, 19).

To our knowledge, there are still no studies that directly compare EA with surgical treatment of recurrent MLNs. Data on the clinical efficacy of EA in areas where no prior lymph node surgery has been performed are still sparse (19).

Although both the *BRAF*^V600E^ mutation and certain aggressive histological subtypes of PTC (diffuse sclerosing, tall cell, columnar cell, solid, and hobnail subtype), have been linked to more aggressive tumor features, no studies have, to our knowledge, investigated the relationship between such aggressive features in the primary tumor and the outcome from EA in MLNs from PTC (9, 22-28).

The effect of the pretreatment volume/size of MLNs on the outcome of EA has been reported in previous studies in which the clinical efficacy is shown to be superior in smaller MLNs (11, 12, 15, 16, 29). However, the effects of other ultrasonographic features on the clinical effects of EA have not been determined.

As a nonsurgical option, it is important to identify patients most likely to benefit from EA, thereby reducing the risk of complications associated with repeated surgeries. This study aimed to explore whether specific ultrasonographic features of the MLN, previous lymph node surgeries in the area, *BRAF*^V600E^–mutated primary tumors, or aggressive subtypes of PTC can serve as predictors to enhance clinical decision-making and patient selection for successful EA outcomes.

## Materials and Methods

The study was approved by the regional committee for medical and health research ethics (REK). Written informed consent was obtained from all included patients. The cause of death for deceased patients was obtained from the Norwegian Cause of Death Registry.

### Inclusion of Patients

**Figure 1.**
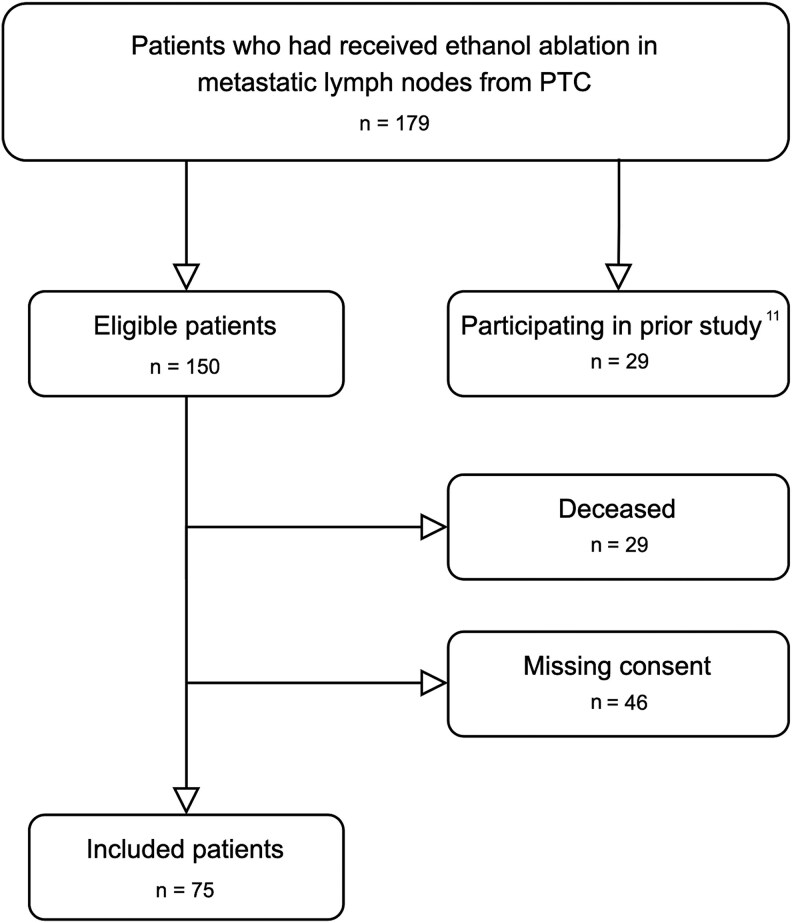
Flowchart of patient disposition.

Procedure codes in the Radiology Information System (RIS) were examined to identify patients who underwent chemical ablations at the Norwegian Radium Hospital, Oslo University Hospital, from 2007 to 2020. We reviewed medical records to identify those treated with EA for MLNs from PTC. Patients from our previous follow-up study were excluded (17), resulting in 150 patients, of whom 29 were deceased, leaving 121 eligible. From those, 75 participated, while 46 did not respond or consent. Patient details are shown in Fig. 1.

### Methods

Patients were classified according to the American Joint Committee on Cancer Tumor Node Metastasis, 8th edition (30).

Follow-up evaluations for the enrolled patients were conducted from February 2021 to January 2023. Data were sourced from medical records and the radiological (RIS/PACS) system. Patients were either called for outpatient reexamination, or data from regular follow-ups after study inclusion were collected. Experienced endocrine surgeons interviewed all patients, either in person or via phone, to discuss medical history and review experiences with EA treatments and side effects. Neck ultrasounds were performed by radiologists with more than 5 years of PTC evaluation experience, using the GE ML6-15-D matrix linear probe with a 4.5- to 15-MHz frequency range on the GE Logiq E9 (GE Healthcare).

We assessed all earlier EA-treated MLNs based on their ultrasound characteristics. Any new lymph nodes suspected of metastasis were recorded. The pretreatment volume of lesions was calculated with the formula for the volume of an ellipsoid (^4^/_3_·π·a·b·c) using the semi-axes of the length (a), width (b), and depth (c) of the MLN. All MLNs were organized, according to their anatomical location, into left/right lateral (level II-V) and central (level I and VI) neck regions (NRs). The outcome of previous EAs and the time to recurrence were evaluated. We considered the outcome of EA treatment in the separate MLN to be successful when one or more of the previously published criteria were met (Table 1) (11, 17). We confirmed loco-regional control (LRC) when no sign of viable MLN was detectable by high-frequency ultrasound. Progressive disease in the neck was defined as tumor progression at the localization of previous ablations or the occurrence of new MLN recurrences elsewhere on the neck. For MLN requiring multiple EA injections, the standard interval between successive EA treatments in our department is 4 to 6 months. Therefore, progression at the ablation site was confirmed only when regrowth was observed more than 9 months after the last EA procedure, to differentiate true progression from temporary regrowth during ongoing EA treatment.

**Table 1.** Satisfactory ethanol ablation was defined as meeting one or more of the listed criteria

Criteria for satisfactory ethanol ablation	
1	Complete lymph node disappearance
2	Reduction of the anteroposterior lymph node diameter to ≤4 mm without visible vascularization
3	Normalization of lymph node size and appearance, and in some nodes scar formation
4	No detectable malignant cells in the FNAB specimens and no detectable Tg in FNAB needle wash

Abbreviations: FNAB, fine-needle aspiration biopsy; Tg, thyroglobulin.

We retrospectively reviewed patient medical records to evaluate prior neck surgeries. Several surgical reports were lacking comprehensive descriptions and exact locations of dissected lymph nodes according to lymph node levels. To achieve accurate data, we classified previous lymph node surgeries into the left/right lateral (levels II-V) and central (levels VI and VII) NRs for further analysis.

Two patients were excluded from the BRAF^V600E^ analysis due to missing paraffin blocks. BRAF^V600E^ protein analysis was performed on tissue samples from the primary tumor in most cases (69/73) and in tissue samples from MLNs in the remaining 4 cases. We excluded 3 patients from analysis of serum thyroglobulin (s-Tg) values due to the presence of Tg antibodies (TgAb). Details of the BRAF^V600E^ immunohistochemical staining technique, the histopathological assessment procedures, and the biochemical analysis methods are provided in a supplementary data file (31).

### Statistics

IBM SPSS Statistics version 29 was used for data analysis. The Fisher exact test and Mann-Whitney *U* test were used to compare categorical variables. Binary logistic regression analysis was performed for multivariable analysis.

## Results

### Patient Population

Out of the 150 eligible patients, 29 were deceased. Of the 121 living patients, 75 were included in this long-term follow-up. There was a slight overrepresentation of female patients (57%). Median age at follow-up was 57 years (range, 31-89 years). All included patients had undergone total or subtotal thyroidectomy in 1 or 2 steps as primary treatment, followed by a single or repeated ^131^I ablation (median 1.5; range, 1-5). Median time from primary thyroid surgery to first EA treatment was 18 months (range, 3-404 months) and median time from primary thyroid surgery to follow-up was 119 months (range, 39-471 months, 1 missing). Baseline patient characteristics are presented in Table 2.

**Table 2.** Baseline patient characteristics

	Included patients		Deceased patients	
	No.	%	No.	%
Sex				
Male	32	42.7	11	37.9
Female	43	57.3	18	62.1
Initial surgery				
Total*^a^*	65	86.7	24	85.7
Hemi*^b^*	—	—	2	7.1
2-Step total*^a^*	10	13.3	2	7.1
Missing			1	
Initial lymph node dissection				
Central	27	38.6	4	14.3
Central + Lateral	24	34.3	14	50.0
Lateral	9	12.9	1	3.6
None	10	14.3	9	32.1
Missing	3		1	
Postoperative ^131^I ablation*^c^*				
0	—	—	2	7.4
1	34	48.6	8	29.6
2	24	34.3	10	37.0
3	7	10.0	3	11.1
4	3	4.3	2	7.4
≥5	2	2.9	2	7.4
Missing	3		2	
Distant metastasis at follow-up or death				
Yes	12	16.4	16	55.2
No	61	83.6	13	44.8
Missing				

^*a*^Total thyreoidectomy.

^*b*^Hemithyreoidectomy.

^*c*^Radioiodine therapy.

TNM status and disease stage at diagnosis of the surviving patients included in the follow-up, as well as the deceased patients, are presented in Table 3. The median age at death for the deceased patients was 78 years (range, 42-91 years). At initial diagnosis, approximately 4 out of 29 (14%) of the deceased patients presented with synchronous distant metastasis (M1), in contrast to none of the included surviving patients (*P* = .001). PTC was identified as the cause of death in 12 of the cases, resulting in cause-specific mortality of 8%. TNM status and stage for patients dying of PTC are shown in Table 4. Median interval from initial surgery to the event of cause-specific death from PTC was 5 years (range, 2-19 years). For patients who died of other causes, the median interval from initial surgery to death was 7 years (range, 2-18 years).

**Table 3.** TNM status*^b^* and disease stage at diagnosis of the surviving patients included in the follow-up, and the deceased patients

	Included patients		Deceased patients		Test of statistical significance*^a^*
	No.	%	No.	%	
Primary tumor					
T1	18	27.3	4	16.0	*P* = .330
T2	14	21.2	7	28.0	*U* = 931
T3	17	25.8	5	20.0	
T4	17	25.8	9	36.0	
Tx	9		4		
Nodal status					
N0	18	26.9	5	18.5	*P* = .397
N1	49	73.1	22	81.5	*U* = 980
Nx	8		2		
Stage					
I	51	70.8	6	23.1	*P* = .001*^c^*
II	8	11.1	12	46.2	*U* = 1361.5
III	11	15.3	3	11.5	
IV	2	2.8	5	19.2	
Missing	3		3		
Distant metastasis					
M0	71	100.0	25	86.2	*P* = .001*^c^*
M1	—		4	13.8	*U* = 1171.5
Missing	4				

^*a*^Mann-Whitney *U* test.

^*b*^American Joint Committee on Cancer Tumor Node Metastasis, 8th edition.

^*c*^Statistical significance level of *P* < .01.

**Table 4.** TNM status and stage for the 12 patients with disease specific death from papillary thyroid carcinoma

	<55 y*^a^*				≥55 y*^a^*			
	T	N	M	No. of patients	T	N	M	No. of patients
Stage 1	4a	1b	0	1	2	0	0	1
Stage 2	4a	1b	1	1	2	1b	0	2
Stage 3					4a	1b	0	3
Stage 4a					4b	1b	0	1
Stage 4b					4b	1a	1	1
					4b	1b	1	2
				2				10

^*a*^Age at primary diagnosis.

### Included Metastatic Lymph Nodes

We included a total of 134 EA-treated MLN in the analysis. Fifty-seven (42.5%) of the lesions were located in the central NR, while the remaining 77 lesions were distributed in the left (33/134) and right (44/134) lateral regions. The median number of EA-treated lesions in each NR was 1 (range, 1-5), and the median number of EA-treated lesions per patient was 1 (range, 1-9). The highest number of EA-treated MLNs within a single NR was 5. Concurrent treatment was limited to a maximum of 3 MNLs within the same NR, except in 1 instance in which 4 MNLs were treated in the same session. Most lesions were solid (110/134) or partially cystic (11/134), leaving a smaller subgroup of pure cystic lesions (13 MLNs). The mean initial volume of the MLNs was 0.35 mL (SD ± 0.63).

### Clinical Efficacy of Ethanol Ablation per Individual Metastatic Lymph Nodes

**Figure 2.**
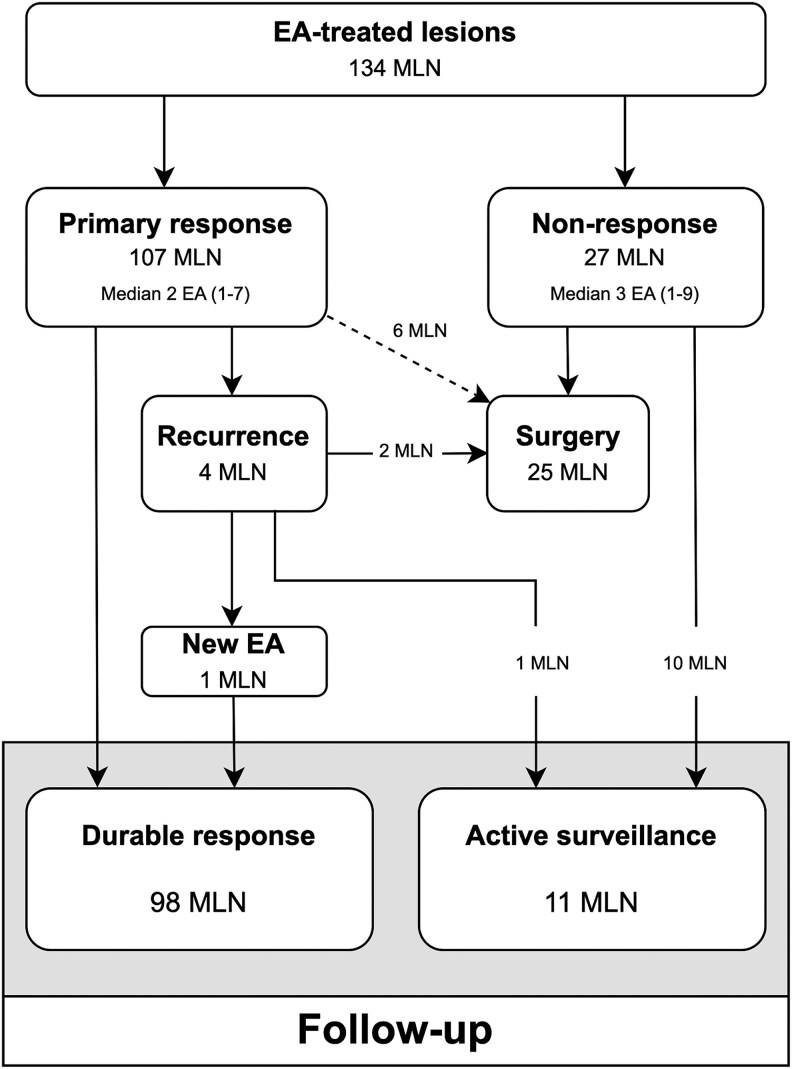
The figure shows the outcome of treatment with ethanol ablation (EA) in the included metastatic lymph nodes (MLNs). Primary response was seen in most (80%) of the EA-treated lesions. Median time from first to last EA in the responding MLNs was 4 months (range, 0-58 months). Active surveillance was chosen for 10 of the nonresponding lesions while the remaining 17 nonresponding lesions were removed by subsequent revision surgery. Recurrence at the ablation site occurred in 3.7% (4/107) of the lesions having primary response to EA. The median time from first EA to the detection of the recurrence was 40.5 months (range, 10-83 months). Most recurring MLNs (3/4) occurred in patients with stage I disease while the last recurrence on the site of ethanol ablation occurred in a patient with stage II disease. One of the recurrent MLNs had durable response from new EA, 2 were surgically removed, and the last recurrent MLN was followed by active surveillance. Six MLNs, which initially responded to EA treatment or were scheduled for additional EA therapy, were excised during subsequent lymph node surgery in the same neck region and thus lost to follow-up.

We found durable response at follow-up in almost three-quarters (98/134, 73%) of the lesions. The outcome of the EA treatment in the individual MLNs are presented in Fig. 2. The median time from the last EA performed to follow-up was 80.5 months (range, 0-139 months). The median number of injections per MLN was 2 (range, 1-9). A single EA procedure was sufficient to achieve a lasting response in 49 out of 98 MLN. The remaining 49 MLNs required repeated EA injections to achieve a durable response, although, in most successfully treated lesions, this was accomplished after 3 or fewer procedures (78/98, 80%). The number of EA procedures (median 2) did not differ between the responding (range, 1-8) and nonresponding lesions (range, 1-9).

**Figure 3.**
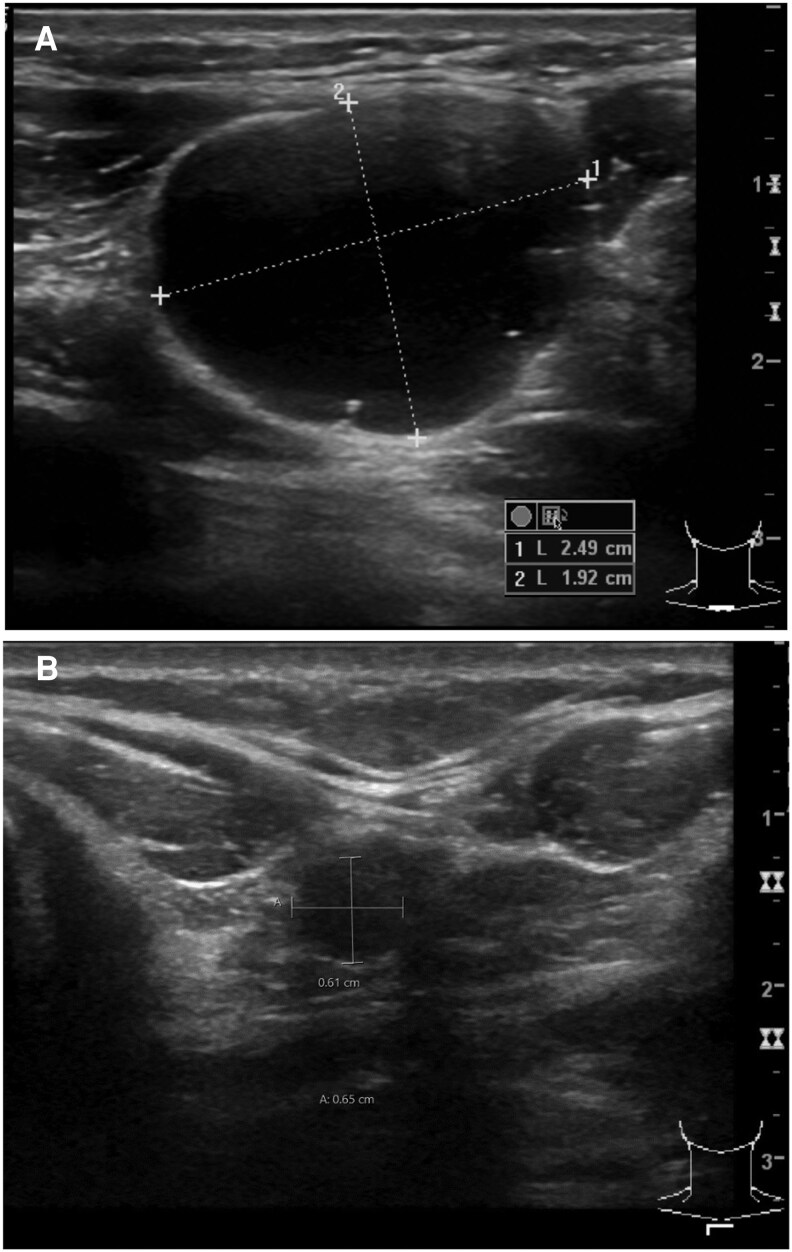
A, A large pure cystic metastatic lymph node (MLN) of papillary thyroid carcinoma in a 37-year-old male patient (T2N1bM0, stage I), measured 25 × 19 × 21 mm (5.2 mL) before ethanol ablation (EA). Initially, the MLN was treated using an injection of 0.6 mL of ethanol. B, Six months later ultrasound (US) examination showed a reduction in size to 6 × 7 × 7 mm (0.15 mL). A second EA was performed, this time injecting a total of 0.3 mL of ethanol. No residual lesion was seen in the following US. Two noncystic MLNs in the same region were also treated by EA; however, neither exhibited a primary response after 9 and 1 injections, respectively. The large cystic MLN (A and B) remained undetectable until it was lost to follow-up 3 years after the initial EA, at which point a neck node dissection was performed to remove the 2 nonresponsive MLNs in the same area. The patient had loco-regional control with no detectable MLN in the neck at follow-up examination 11 years (133 months) after primary surgery.

Pure cystic lesions had a favorable outcome compared to solid or partially cystic lesions, with an excellent response rate regardless of initial size (100% vs 74%; *P* = .039). An example of a successful EA treatment of a large (5.2 mL) pure cystic MLN is given in Fig. 3. We did find a statistically significant difference in the response rate between smaller lesions (≤0.5 mL) and larger lesions (>0.5 mL) when pure cystic lesions were excluded (77% vs 53%; *P* = .045) (Table 5). Multivariable analysis of the predictive factors for response in EA-MLN showed a similar but not statistically significant trend (odds ratio [OR] 0.814-8.155; *P* = .107) (Table 6).

**Table 5.** Univariable analysis—response in metastatic lymph nodes treated with ethanol ablation

Factor	Group	No. of MLNs	Response		*P^a^*
			Yes	No	
Stage					
	I-II	104	81	23	.609
	III-IV	26	19	7	
	Missing	4			
Subtype					
	Classic	100	75	25	.099
	Aggressive*^b^*	24	22	2	
	Missing	10			
*BRAF* ^V600E^					
	Negative	25	17	8	.181
	Positive	99	80	19	
	Missing	10			
Initial volume*^c^*					
	≤0.5 mL	92	71	21	.045*^d^*
	>0.5 mL	19	10	9	
	Missing	10			
Cystic					
	Solid/partially cystic	121	90	31	.039*^d^*
	Pure cystic	13	13	0	
	Missing	—			

MLNs that were cystic exhibited a significantly more favorable response rate than those that were either solid or partially cystic, with all cystic MLNs demonstrating a lasting response. Lesions initially measuring less than 0.5 mL in volume showed a significantly superior response rate compared to those with volumes exceeding 0.5 mL. The disease stage at the time of diagnosis, a positive *BRAF*^V600E^ status, or an aggressive subtypes of the primary tumor did not significantly affect the response rate from EA in the individual MLNs.

Abbreviations: EA, ethanol ablation; DSV, diffuse sclerosing variant; MLN, metastatic lymph node; TCV, tall cell variant.

^*a*^Fisher exact test.

^*b*^TCV and DSV variants.

^*c*^Pure cystic lesions (13 MLNs) were excluded.

^*d*^Statistical significance level of *P* less than .05.

**Table 6.** Multivariable analysis of predictive factors for having response in EA-treated metastatic lymph nodes

Factor	Group	No. of MLNs	Response		OR	OR 95% CI	*P^a^*
			Yes	No			
Stage							
	I-II	104	81	23	1.717		
	III-IV	26	19	7		0.529-5.575	.369
	Missing	4					
Subtype							
	Classic	100	75	25	0.258		
	Aggressive*^b^*	24	22	2		0.050-1.323	.104
	Missing	10					
*BRAF* ^V600E^							
	Negative	25	17	8	0.768		
	Positive	99	80	19		0.237-2.491	.661
	Missing	10					
Initial volume*^c^*							
	≤0.5 mL	92	71	21	2.577	0.814-8.155	.107
	>0.5 mL	19	10	9			
	Missing	10					

Multivariable analysis of the investigated predictive factors did not show statistically significant differences in response rates following EA. A nonsignificant trend toward a higher response rate to EA was seen in lesions measuring 0.5 mL or less.

Abbreviations: EA, ethanol ablation; DSV, diffuse sclerosing variant; MLN, metastatic lymph node; OR, odds ratio; TCV, tall cell variant.

^*a*^Wald chi-square test.

^*b*^TCV and DSV variants.

^*c*^Pure cystic lesions (13 MLNs) were excluded.

As shown in Table 5, there was no statistically significant difference in the univariate analysis comparing the response to EA in MLNs with or without the presence of the *BRAF*^V600E^ mutation in the primary tumor (81% vs 68%; *P* = .181). An aggressive pathological subtype was confirmed in the primary tumor of 12 patients representing 24 of 134 MLNs (20 tall cell subtype, 2 diffuse sclerosing subtype, and 2 mixed tall cell and solid subtype). There were no statistically significant differences in the response rate to EA in the MLNs in which an aggressive subtype had been confirmed in the primary tumor compared to patients with classic subtypes (see Table 5).

### Clinical Efficacy of Ethanol Ablation per Patient

Approximately two-thirds (57/75) of the patients had achieved LRC with no detectable disease on the neck on their follow-up ultrasound. LRC on the neck had been achieved after 1 or more revision surgery procedures in 13 of these patients, while LRC on the neck was achieved solely by EA in the remaining 44 patients. The latter group members are referred to as “good EA responders” in the following. At follow-up, 15 of 75 patients had known distant metastasis from PTC; 2 of these patients belonged to the group of good EA responders. We conducted a multivariable analysis to identify predictive factors among good EA responders vs patients without LRC or requiring further surgery (Table 7). Patients with 3 or more MLNs treated with EA were significantly more likely to require subsequent surgery or have persistent MLN in the neck at follow-up (OR 6.945; 95% CI, 1.422-33.922; *P* = .017). As anticipated, lower disease stages (I and II) correlated with improved treatment outcomes from EA, compared to stages III and IV (OR 30.510; 95% CI, 2.866-324.740; *P* = .005). Nevertheless, the presence of aggressive characteristics in the primary tumor did not statistically significantly affect the outcome.

**Table 7.** Multivariable analysis of predictive factors for being a “good ethanol ablation responder”

Factor	Group	No. of patients	Good responder		OR	OR 95% CI	*P^a^*
			Yes	No			
Stage							
	I-II	71	39	19	30.510		
	III-IV		2	11		2.866-324.740	.005*^c^*
	Missing	4					
Subtype							
	Classic	69	33	24	0.138		
	Aggressive*^b^*		8	4		0.014-1.394	.093
	Missing	6					
*BRAF* ^V600E^							
	Negative	69	9	7	1.075		
	Positive		32	21		0.260-4.453	.921
	Missing	6					
No. of EA-MLN							
	1-2	75	40	24	6.945	1.422-33.922	
	≥3		4	7			.017*^d^*

Patients classified as “good EA responders” were those who attained locoregional control in the neck through EA alone, with no need for revision surgery. Individuals with lower disease stages (I and II) at diagnosis, and having fewer than 3 MLNs treated with EA (EA-MLN), had a statistically significantly higher probability of being “good EA-responders.” The outcome was not significantly influenced by the presence of aggressive characteristics in the primary tumor.

Abbreviations: EA, ethanol ablation; DSV, diffuse sclerosing variant; MLN, metastatic lymph node; OR, odds ratio; TCV, tall cell variant.

^*a*^Wald chi-square test.

^*b*^TCV and DSV variants.

^*c*^Statistical significance level of *P* < .01.

^*d*^Statistical significance level of *P* < .05.

### Biochemical Analysis

Almost all (41/42) the good EA responders without evident distant metastasis at follow-up had s-Tg-values of less than 1.0 μg/L, of whom most (68.3%) had s-Tg-values of less than 0.01 μg/L. The last patient had a moderately elevated s-Tg (2.8 μg/L).

About two-thirds (9/13) of the patient group for which LRC was achieved after 1 or more revision surgery procedures had no evident distant metastasis at follow-up. All of these patients had had s-Tg values of less than 1.0 μg/L, of whom 6 (66.7.2%) had s-Tg-values of less than 0.01 μg/L.

All except 1 (13/14) of the patients with evident distant metastasis at follow-up had elevated s-Tg (range, 0.5-66.0). The last patient had received iodine radiotherapy for multiple lung metastasis 15 months before follow-up in this study. Despite a recent chest computed tomography scan showing progression of the lung metastases, s-Tg levels were undetectable (s-Tg <0.10 μg/L, TgAb 44 kIU/L).

### Outcome of Ethanol Ablation in Neck Regions With or Without Prior Metastatic Lymph Node Surgery

**Figure 4.**
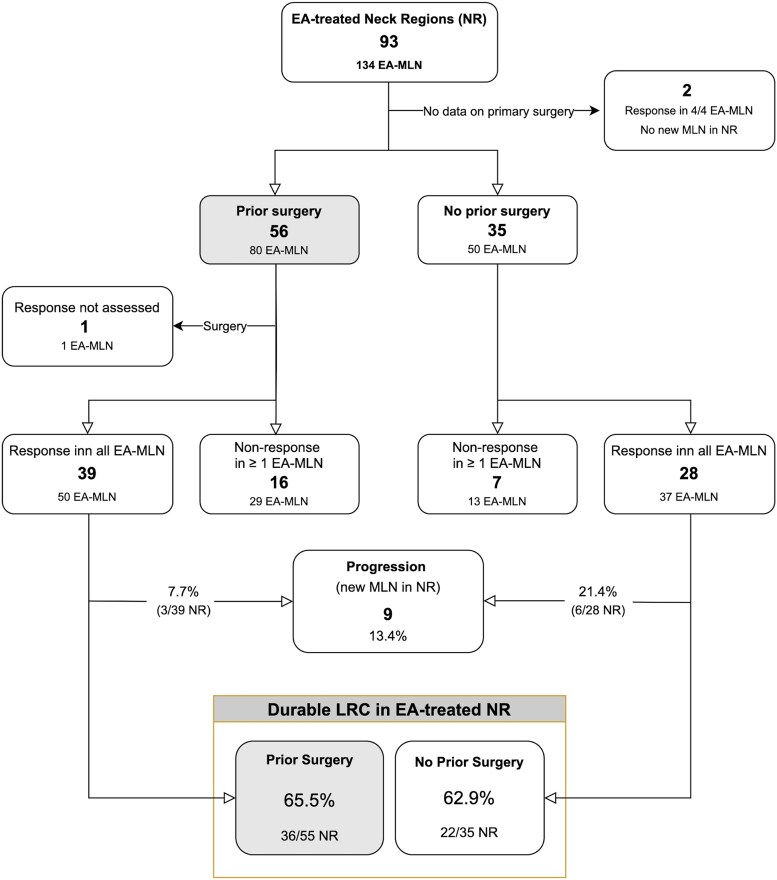
EA was performed in 134 metastatic lymph nodes (EA-MLN) in 93 lateral or central neck regions (NRs) in the 75 papillary thyroid carcinoma patients who were included in the study. Two NRs (4 EA-MLN), in 2 patients, were excluded from the analysis due to missing information on previous surgeries. One EA-MLN (1 NR) was excised surgically before the treatment effect of EA could be evaluated. The number of MLN treated was similar in the NR with and without prior surgery (median 1, range 1-5). Most NRs where EA was given as upfront treatment had single-node disease (66%, 23/35). With 1 exception, no more than 2 lesions were treated with EA within the same nonoperated NR. In the last case the patient was treated in 5 MLNs in the same NR over a course of more than 6 years, during which 80% (4/5) of the lesions showed a lasting response at follow-up. Progression outside the localizations of EA occurred in 13% of the NRs. Loco-regional control (LRC) with no detectable MLN in the NR was confirmed in almost two-thirds of the EA-treated NRs. There was no statistically significant difference in the rate of LRC between NRs with and without lymph node surgery prior to EA (*P* = .825).

Although surgical resection is typically considered the standard treatment for managing MLN, EA was performed in one or more nonoperated NRs in a considerable proportion of the included patients (31/75, 41%), based on clinical experience and institutional practices. Multidisciplinary tumor board meetings were not conducted routinely, but convened for 13 of 31 of the patients due to uncertainty by the attending surgeon regarding the optimal treatment strategy. The decision to offer EA in the 35 previously nonoperated NRs was due to small volume disease or lesions being predominantly cystic in 25 NRs (71%), comorbidities in 5 NRs (14%), and heightened risk of complications from surgery in 2 NRs (6%). The patient refused surgery in 3 NRs (9%). As shown in Fig. 4, primary response was achieved in all EA-treated MLNs in 70% (39/56) of the NRs in which lymph node surgery had been performed and in 80% (28/35) of the NRs not preceded by lymph node surgery (*P* = .334). The durable LRC rates in the individual NRs did not show statistically significant differences between the two groups (66% vs 63%; *P* = .825). We found a higher rate of new lesions outside the ablation sites in NRs that had not undergone prior surgery (21%) compared to NRs in which surgery had been performed (8%), but the difference was not statistically significant (*P* = .149). Following a median follow-up period of 122 months (range, 49-471 months) from the initial PTC diagnosis, the incidence of distant metastases in patients who received EA in one or more NRs without prior lymph node surgery (10/25) was not significantly different from the incidence in those who had EA only in NRs in which previous lymph node surgery had been performed (5/29) (22% vs 17%; *P* = .769).

### Complications from Ethanol Ablation

No major complications from EA occurred in the study group. Transient postprocedure hoarseness, attributed to ethanol leakage affecting the laryngeal nerve, was observed in 6.7% (5/75) of the patients. One of the patients was referred for laryngoscopy, but the results from the examination were not available. Laryngoscopy was not conducted for the remaining 4 patients. The hoarseness resolved spontaneously in all patients within 3 months. Pain and discomfort associated with the procedure occurred relatively frequently (30/75 patients), but were transient in all cases.

## Discussion

Durable response after EA of MLNs was achieved in all pure cystic lesions, independent of pretreatment size. The response rate in the pure cystic MLNs was superior to the response rate seen in solid and partially cystic lesions. This difference was statistically significant. This effect may be similar to the well-documented superior treatment response when EA is used to treat benign cystic lesions in the thyroid gland, achieving volume reductions of 85% to 95% (32, 33). The superior response to treatment effect in pure cystic lesions is probably the result of a more complete distribution of the injected ethanol in the lesions, as well as a relatively smaller volume of viable tumor cells, compared to the solid and partially cystic metastases.

It has been proposed that EA is more suitable for treating small metastatic lesions, with some suggesting that EA should be reserved for lesions with a greatest diameter of less than 2 cm (34). The response rate after EA in solid or partially cystic lesions was consistent in lesions up to 0.5 mL, whereas a lower response rate was achieved in lesions with an initial volume greater than 0.5 mL. Although this difference was significant in the univariate analysis, the multivariable analysis, adjusted for other parameters, revealed only a nonsignificant trend. Thermal ablation methods, including radiofrequency, laser, and microwave ablations, are well established as minimally invasive approaches for managing MLNs in PTC. Studies indicate that thermal ablation is both an effective and safe treatment option for larger MLNs, up to 20 mm in diameter (35, 36). Therefore, these techniques would be more appropriate than EA for larger lesions, due to their superior ability to achieve more comprehensive ablation. While the results suggest that EA might be best suited for cystic and smaller solid MLNs, more than half of the lesions in this study with volumes greater than 0.5 mL did show a sustained response. This suggests that EA might still be worth considering in specific cases, especially if thermal ablation options are not available. If EA is chosen for larger lesions, alternative treatment options such as thermal ablation, surgery, or surveillance should always be considered if the desired response is not achieved after 2 or 3 EA sessions.

We found that the efficacy of EA in individual MLNs was comparable to previously published results, with a durable response in 73% of the EA-treated MLN (12, 16, 17, 19, 21, 34). The probability of achieving LRC by EA, without the need for additional surgery, was significantly lower in patients with advanced disease stages (III and IV) and when more than 3 MLN had been ablated. These findings align with previous research and support current guidelines restricting EA to instances involving a limited number of recurrent MLNs in the neck (9, 37).

The rate of recurrences at the ablated sites was lower than we expected, with 3.7% compared to 12% to 24% reported in recent studies (12, 16, 21). The criteria for a treatment response in ablated MLNs varies in different publications and can therefore influence the reported recurrence rates. However, the observed recurrence rate in this manuscript was also lower compared to the prior publication by our own institution (19%), for which similar response criteria as in this study were used (17). There has been a shift in our clinical practice in recent years implementing a lower threshold for a crossover to surgery in cases for which the desired treatment effect is lacking after the initial 1 to 3 EA sessions. The surgical removal of these lesions may have contributed to a lower recurrence rate in this manuscript, but this effect could not be confirmed. Although some concerns have been raised about the possibility of late recurrences after EA in recent studies, the low recurrence rate found in our research is in line with the results presented in a recent study by Hay et al (8). In their study, no signs of regrowth were observed in 71 MLNs treated with EA after a median follow-up of 14 years. To verify a successful EA in their study, both a decrease in the size of the lesion and a complete absence of vascularization on Doppler ultrasound were required.

According to current guidelines and established clinical practice, EA is considered an alternative treatment to surgery in selected patients with limited recurrent MLNs who cannot undergo additional radioiodine therapy and are either poor candidates for surgery or refuse surgical intervention (9, 37). Currently, aggressive characteristics of the primary tumor are not included in any clinical guidelines for decision-making to determine which patients are most likely to benefit from EA. To our knowledge this is the first study to investigate the relationship between aggressive tumor markers, that is, histological subtype and a positive *BRAF*^V600E^ mutation in the primary tumor, and the outcome from EA in patients with persistent or recurrent MLNs from PTC. As these factors are associated with higher rates of recurrences and metastases, a hypothesis is that patients having tumors with these characteristics are less suitable candidates for minimally invasive treatments such as EA (23, 24, 26 , 27, 38). We did not find that a positive *BRAF*^V600E^ mutation status or an aggressive histological subtype in the primary tumor inflicted on the treatment outcome from EA. The treatment response to EA in individual MLNs in the neck from primary tumors that had an aggressive subtype or positive *BRAF*^V600E^ mutation status was not inferior compared to MLN from nonaggressive or *BRAF*^V600E^–negative tumors (see Table 5). Furthermore, we achieved LRC of the neck solely from EA in almost two-thirds (59%) of the patients, independent of subtype or positive *BRAF*^V600E^ mutation status.

Our results suggest that a neither a positive *BRAF*^V600E^ mutation status nor an aggressive histological subtype in the primary tumor is crucial to determine if the patient will be a suitable candidate for EA.

Data on the use of EA as a treatment alternative in NRs without prior lymph node dissection remain limited (9, 37). We found that durable LRC was achieved in the majority of cases also where EA was used in NRs that had not undergone previous surgical treatment. These findings align with a recent publication reporting no significant differences in recurrence rates between patients who had undergone lymphadenectomy prior to EA and those who had not (19). Given the small number of patients and the limited extent of disease, the results should be interpreted with caution. While surgery remains the first-line treatment, further evidence is needed to better understand any potential role of EA as an alternative to surgery in selected cases, also in surgically untreated NRs.

### Limitations

The indolent nature of PTC, characterized by its slow progression and low mortality rates, coupled with the wide array of treatment options, complicates the design of clinical studies to evaluate the effectiveness of specific treatments.

Many eligible patients were not included in the study, which increases the likelihood of selection bias and potentially affects the generalizability of the findings. The limited sample size, and low statistical power from dividing data into small subgroups, requires some caution in the interpretation of the results. Most of the patients in the study had been followed up at our institution, and most of the data were accessible from local medical records and radiological archives. However, the retrospective design of the study, and the long follow-up periods for many of the patients since their initial diagnosis, pose a considerable risk of information bias due to incomplete or inaccurate documentation.

Due to the retrospective nature of the study, including a considerable number of old cytology specimens from fine-needle aspirations, it was impossible to analyze BRAF^V600E^ mutations in the EA-treated lymph nodes. Past research has shown a correlation between *BRAF*^V600E^ mutations in primary tumors and lymph node metastases in the neck. Vasko et al (39) reported that 81% of MLNs in patients with a *BRAF*^V600E^–positive primary tumor exhibited a *BRAF*^V600E^ mutation. Although the status of the *BRAF*^V600E^ mutation in the MLNs treated with EA remains undetermined, we assumed that most of the MLNs from *BRAF*^V600E^–mutated primary tumors treated with EA would likely have expressed the *BRAF*^V600E^ mutation.

This study did not include genetic analysis beyond BRAF^V600E^ immunohistochemistry. The status of *BRAF*^V600E^ mutation as an independent prognostic marker in PTC remains debatable and has not yet been validated by current research (22-26, 40). Numerous reports in the literature propose that incorporating multiple genetic analyses, including telomerase reverse transcriptase (*TERT*) promoter mutations, could enhance the accuracy of predicting treatment outcomes. Incorporating *TERT* or other molecular markers in this study would probably have offered further prognostic insights in EA treatments.

### Summary

A high response rate was found in pure cystic neck node metastases, making EA a superb minimally invasive treatment option. Consistent with previous findings, a lower response rate to EA was found in larger, solid, or partially cystic MLNs. However, a lasting response was observed also in a substantial number of MLNs larger than 0.5 mL. There was no correlation between positive *BRAF*^V600E^ mutation status and aggressive histological subtype in the primary tumor and the effectiveness of EA in the MLNs. Further research is needed to assess any potential role of EA in NRs without prior lymph node surgery.

## Data Availability

Some or all data sets generated and/or analyzed during the current study are not publicly available but are available from the corresponding authors on reasonable request.

## Abbreviations

EA: ethanol ablation
LRC: loco-regional control
MLN: metastatic lymph node
NR: neck region
OR: odds ratio
PTC: papillary thyroid carcinoma
s-Tg: serum thyroglobulin
TERT: telomerase reverse transcriptase
TgAb: thyroglobulin antibodies
